# A study of otolith function in patients with orthostatic dizziness

**DOI:** 10.1007/s00405-023-07985-z

**Published:** 2023-04-28

**Authors:** Nada Medhat El Medany, Enaas Ahmad Kolkaila, Trandil Hassan El Mehallawi, Reham Mamdouh Lasheen

**Affiliations:** 1 Audio-Vestibular Medicine Department, Faculty of Medicine, Tanta University, Tanta, Egypt; 2 Audiovestibular Unit, Department of Otolaryngology/Head and Neck Surgery, Tanta University Hospital, El-Geish Street, Tanta, 31511 El-Gharbia Egypt

**Keywords:** Orthostatic dizziness, Vestibular-evoked myogenic potentials, Subjective visual vertical, Subjective visual horizontal

## Abstract

**Background:**

Orthostatic dizziness (OD) is the dizziness that occurs when moving from a sitting or a supine to a standing position. It is typically thought to be connected to orthostatic hypotension (OH). The otolithic control of respiratory and cardiovascular system through vestibulosympathetic reflex has been the focus of considerable recent interest. This study aimed to evaluate the relationship between the orthostatic dizziness and otolith organ function.

**Methods:**

This study was carried on 50 adults aged from 18 to 50 years with normal peripheral hearing. Subjects were divided into two groups: controls (GI): 20 healthy adults and study group (GII): 30 patients who were complaining of OD. Patients were submitted to; blood pressure measurement in sitting and standing positions, combined vestibular-evoked myogenic potentials (VEMPs) and subjective visual vertical and horizontal tests (SVV) and (SVH).

**Results:**

The study group showed abnormal absent cVEMP, oVEMP. There were also statistically significant differences of P13 and N23 latencies and (P13N23) amplitudes between the two groups in the left ears. Both groups differed significantly in SVH values deviated to the left side. Study group were further subdivided into ten patients with OH and 20 patients with OD without OH. The both study subgroups showed abnormal absent cVEMP, oVEMP and abnormal SVH. OH patients showed statistically significant differences of cVEMP waves P13, N23 latencies in the left ears when compared with the control.

**Conclusions:**

Otolith malfunction may be the cause of orthostatic dizziness (OD) in patients with and without orthostatic hypotension.

## Introduction

When someone has dizziness or vertigo exclusively when standing up straight from sitting or lying down positions, they are said to be suffering from orthostatic dizziness (OD) [1].

Orthostatic hypotension (OH) is characterized by a prominent reduction in systolic (≥ 20 mmHg) and/or diastolic (≥ 10 mm Hg) blood pressure within 3 min of standing or during a head-up tilt test [2]. Of the many potential causes of orthostatic dizziness/vertigo is OH, but it is not the only cause.

One of the organs that sense gravity, the otolith, it is able to send a signal to the brain if there is a sudden change in the body's position in relation to the gravity. Evidence suggests that problems with the otolith organ and the vestibulo-sympathetic reflex may contribute to the onset of orthostatic dizziness [3].

The aim of this study was to evaluate the relationship between the orthostatic dizziness and otolith organ function using combined vestibular-evoked myogenic potentials (VEMPs) and subjective visual vertical/horizontal tests.

## Patients and methods

This study was carried out on 50 adults with normal peripheral hearing. Their age ranged from 18 to 50 years. This study was approved by the Research Ethical Committee of Faculty of Medicine, Tanta University with approval code 32828/01/19. Subjects with neurological problems, cardiac diseases, general health problems, neck or visual problems (apart from standard refractive errors) and subjects with hearing loss were excluded from this study. Participants were divided into two groups: 20 healthy adults made up the controls (GI) and Study group (GII) included 30 patients who have OD with no complaint regarding hearing.

All the patients subjected to full detailed history taking about the character, the inducing factors, duration, frequency and associated symptoms of their dizziness, to exclude different causes of dizziness. None of the patients was complaining of Benign paroxysmal positional vertigo (BPPV), as the patient was asked if the vertigo precipitated by sudden head movement, turning in bed, looking upward or downward. Blood pressure measurement was done in sitting and within 3 min upon standing. The reduction should be ≥ 20 mmHg systolic and/or ≥ 10 mmHg diastolic blood pressure to confirm the diagnosis of (OH).

Furthermore, otological examination and basic audiological evaluation were done. These included; Pure tone audiometry for air conduction in the frequency range of 250–8000 Hz and bone conduction in the frequency range of 500–4000 Hz. Speech audiometry including both: Speech Recognition Threshold (SRT) tests using Bisyllabic Words for adults [4] and Word Discrimination score (WD) test using Phonetically Balanced Words for adults [5].

### Combined vestibular-evoked myogenic potentials (VEMPs)

Combined vestibular-evoked myogenic potentials test was performed by Smart EPs of Intelligent hearing system (HIS). For recording cervical VEMPs: two active electrodes, one on each side, were put on the middle of the contracted sternocleidomastoid muscle, and two reference electrodes were inserted on the middle third of both clavicles. Over the patient's forehead, an electrode was put as a grounding source. While for ocular VEMP recoding, two active electrodes were positioned slightly below the lower eyelid. In addition, two reference electrodes were positioned around 1–2 cm below the corresponding active electrodes below each of the eyes. The patient was directed to stare upward, roughly 30 degrees above horizontal, at a distant object in the midline while rotating his or her head to the opposite side of recording and flexing the head approximately 30° forward to contract the SCM. Stimulation of right ear was applied during right cervical VEMPs and left oVEMP recording and vice versa. Stimulus parameters: air conducted alternative 500 Hz tone burst stimuli were presented via an insert phones at 95dBnHL with repetition rate was 5/sec.

For recording the responses: the filter bandpass settings was set 30–3000 Hz. Responses were averaged over 128 sweeps. The time window was set from 50 to 100 ms and 50.000 gain factor was used.

Analysis of the waves: from all recorded traces, the positive and the negative peaks were identified according to their latencies (P13, N23 in cVEMPs) (N10, P15 in oVEMPs). Then, measuring the amplitude of each wave from the positive peak to the negative trough (P13–

N23) in cVEMP waves and (N10–P15) in oVEMP waves.

### Subjective visual vertical and horizontal tests (SVV) and (SVH)

Subjective Visual Vertical and Horizontal Tests was performed by (Multitest FRAMIRAL) connected to projector (Sony). The used device projected a laser red bar on a white flat screen situated in front of the patient one and a half meter away. The light bar was tilted up to approximately 30° from the true vertical/horizontal right side down (clockwise) and left side down (counterclockwise) 3 times on each side. On each time, the subject was asked to rotate the bar to the position that he/she felt was vertical/horizontal using a remote hand controller.

The test was terminated when the examinee informed that he/she had finished the adjustment. Six measurements were taken for each subject for each tests (SVV/SVH). No time limit was used when performing the test. After testing, the mean value of the six trials was calculated as the subject’s score.

### Statistical analysis

All data were analyzed by SPSS version 22, IBM, Armonk, NY, United States of America. Continuous data were tested for normality by Shapiro Wilk test and the P value was greater than 0.05. The data were normally distributed. Quantitative data were expressed as mean ± standard deviation (SD) and percentage. *t* test was used for the comparison between the control and the study group. One-way analysis of variance (ANOVA) and Post Hoc test were used for the comparison between the control and the two study subgroups. Chi-square (*X*^2^) test of significance was used to compare proportions between the two qualitative parameters.

## Results

This study was carried out on 50 adults aged from 18 to 50 years. They all had bilateral normal peripheral hearing in the frequency range of 250–8000 Hz (hearing threshold levels were ≤ 25 dBHL). Participants were divided into two groups: 20 healthy subjects made up the controls (GI) who had neither auditory nor vestibular complaints. The study group (GII): 30 patients who were complaining of OD. There were no significant variations as regard gender and age in the two tested groups (*P* value = 0.642, 0.212), respectively. Moreover, PTA thresholds average and SRT did not differ significantly between the two groups Diastolic BPs in sitting position, systolic and diastolic BPs in standing position were significantly lower in the study group compared to the controls (*P* = 0.038, *P* = 0.004, *P* = 0.010), respectively (Table 1).

**Table 1:** Comparison between the control and the study group as regard blood pressure measurements results

	Range	Mean ± SD	*T* value	*P* value
Systolic sitting				
Control	100–130	116.00 ± 9.81	0.842	0.404
Study	90–130	113.00 ± 13.75		
Diastolic sitting				
Control	60–90	78.10 ± 10.08	2.133	0.038*
Study	60–90	72.50 ± 8.38		
Systolic standing				
Control	90–130	114.00 ± 12.20	3.032	0.004*
Study	80–130	105.00 ± 14.08		
Diastolic standing				
Control	60–90	77.50 ± 9.67	2.679	0.010*
Study	50–90	70.33 ± 9.00		

*Significant at *P* < 0.05

While, Cervical and Ocular vestibular-evoked potential waves were present in all subjects of the controls. In the study group, 17 patients (56.7%) showed absent cVEMPs and 22 patients (73.3%) showed absent oVEMPs. As regard detectability of cVEMPs and oVEMPs waves, there was a significant difference between the control and study groups (*P* = 0.001) (Table 2). All subject in the control group showed normal SVV and SVH, while 4 patients (13%) showed abnormal SSV results and 10 patients (33.3%) showed abnormal SVH results. SVV results were not statistically significant different in the two studied groups. However, both groups differed significantly in SVH values deviated to the left side (*P* = 0.016) (Table 2).

**Table 2:** Detectability of cVEMPs, oVEMPs and percentage of normal and abnormal SVV, SVH in the control and the study group

c VEMP	Control	Study	Total	*X*^2^	*P* value
Present	20 (100.0%)	13 (43.3%)	33 (66.0%)	17.172	0.001*
Absent					
Right	0 (0.0%)	0 (0.0%)	0 (0.0%)		
Left	0 (0.0%)	6 (20.0%)	6 (12.0%)		
Bilateral	0 (0.0%)	11 (36.7%)	11 (22.0%)		
Total	20 (100.0%)	30 (100.0%)	50 (100.0%)		
oVEMP					
Present	20 (100.0%)	8 (26.7%)	28 (56.0%)	26.190	0. 001*
Absent					
Right	0 (0.0%)	0 (0.0%)	0 (0.0%)		
Left	0 (0.0%)	6 (20.0%)	6 (20.0%)		
Bilateral	0 (0.0%)	16 (53.3%)	16 (53.3%)		
Total	20 (100.0%)	30 (100.0%)	50 (100.0%)		
SVV					
Normal	20 (100.0%)	26 (86.7%)	46 (92.0%)	2.902	0.235
Abnormal					
Right	0 (0.0%)	1 (3.3%)	1 (2.0%)		
Left	0 (0.0%)	3 (10.0%)	3 (6.0%)		
Total	20 (100.0%)	30 (100.0%)	50 (100.0%)		
SVH					
Normal	20 (100.0%)	20 (66.7%)	40 (80.0%)	8.329	0.016*
Abnormal					
Right	0 (0.0%)	3 (10.0%)	3 (6.0%)		
Left	0 (0.0%)	7 (23.3%)	7 (14.0%)		
Total	20 (100.0%)	30 (100.0%)	50 (100.0%)		

Data are represented by number (%)

*Statistically significant at *P* ≤ 0.05

*cVEMP* Cervical Vestibular-Evoked Myogenic Potentials, *oVEMP* Ocular Vestibular-Evoked Myogenic Potentials, *SVV* subjective visual vertical, *SVH* subjective visual horizontal

There were statistically significant differences of cVEMP waves P13, N23 latencies and (P13N23) amplitudes between the two groups in the left ears only (*P* = 0.013, *P* = 0.030, P = 0.047), respectively (Table 3). However, there were no significant difference between the two groups as regard oVEMP waves N10, P15 latencies (*P* = 0.929, *P* = 0.331) in right ears, (*P* = 0.892, *P* = 0.889) in left ears, respectively, or (N10–P15) amplitudes (*P* = 0.416, *P* = 0.898) in right and left ears, respectively.

**Table 3:** Comparison of the cVEMPs wave latencies (in msec) and amplitudes (in μv) between control and study group

c VEMP	Range	Mean ± SD	*T* value	*P*. value
Right P13 latency				
Control	10.7–18.5	13.82 ± 1.79	1.422	0.163
Study	11.5–20.9	14.92 ± 2.94		
Right N23 latency				
Control	17.2–24.3	20.80 ± 1.77	1.999	0.053
Study	18.4–28.8	22.30 ± 2.82		
P13–N23 amplitude				
Control	18.1–62.5	37.24 ± 12.85	1.305	0.200
Study	9.2–71.1	31.27 ± 15.67		
Left P13 latency				
Control	11.8–18.3	14.53 ± 1.78	2.630	0.013*
Study	12.2–21.4	16.48 ± 2.49		
Left N23 latency				
Control	17.3–24.2	21.28 ± 1.85	2.269	0.030*
Study	18.7–29.4	23.03 ± 2.58		
P13–N23 amplitude				
Control	18.4–70.4	38.43 ± 13.47	2.071	0.047*
Study	14.1–55.4	28.44 ± 13.65		

*cVEMP* Cervical Vestibular-Evoked Myogenic Potentials

*Significant at *P* < 0.05

Ten patients (33.3%) of the study group were fulfilling the criteria of orthostatic hypotension.

So, the study group (GII) was further subdivided into two subgroups based on their blood pressure testing:

- Subgroup (GIIa) OD with OH (10 patients): in which subjects were complaining from orthostatic dizziness and had orthostatic hypotension.
- Subgroup (GIIb) OD without OH (20 patients): they were complaining from orthostatic dizziness and had no OH.

Comparing the blood pressure measurement between the control and the two study subgroups showed significant difference between the control and OH study subgroup in systolic and diastolic standing BPs (*P* = 0.015, 0.016), respectively. There was also significant difference between the control group and OD without OH subgroup in diastolic standing BPs (*P* = 0.039) (Table 4).

**Table 4:** Comparison between the control and the two study subgroup as regard blood pressure measurements results

	Range	Mean ± SD	*F*. test	*P*. value		
Systolic sitting						
Control	100–130	116.00 ± 9.81	1.381	0.261	P1	0.752
OD with OH	100–130	117.50 ± 10.87			P2	0.180
OD without OH	90–130	110.75 ± 14.71			P3	0.160
Diastolic sitting						
Control	60–90	78.10 ± 10.08	2.449	0.102	P1	0.253
OD with OH	60–90	74.00 ± 8.43			P2	0.052
OD without OH	60–90	71.75 ± 8.47			P3	0.529
Systolic standing						
Control	90–130	114.00 ± 12.20	3.415	0.041*	P1	0.015*
OD with OH	90–120	101.00 ± 9.94			P2	0.103
OD without OH	80–130	107.00 ± 15.59			P3	0.251
Diastolic standing						
Control	60–90	77.50 ± 9.67	3.849	0.028*	P1	0.016*
OD with OH	60–80	68.50 ± 8.18			P2	0.039*
OD without OH	50–90	71.25 ± 9.44			P3	0.449

*GIIa* study subgroup had OD with OH, *GIIb* study subgroup had OD without OH, *P1* Control and OD with OH, *P2* Control and OD without OH, *P3* OD with OH and OD without OH

*Significant *p* value < 0.05

In OH study subgroup (IIa), four patients (40%) showed bilateral absent cVEMP waves. While eight patients (80%) showed absent oVEMP waves either bilaterally or in the left ears. Furthermore, five patients showed abnormal SVH values (50%) and only one patient showed abnormal SVV values (10%) (Table 5).

**Table 5:** Comparison of detectability of cVEMPs, oVEMPs waves and percentage of normal and abnormal SVV, SVH between the control and both study subgroups

c VEMP	Control	GIIa	GIIb	*P*. value
Present	20 (100%)	6 (60%)	7 (35%)	0.001*
Absent				
Rt	0 (0%)	0 (0%)	0 (0%)	
Lt	0 (0%)	0 (0%)	6 (30%)	
Bilateral	0 (0%)	4 (40%)	7 (35%)	
o VEMP				
Present	20 (100%)	2 (20%)	6 (30%)	0.001*
Absent				
Rt	0 (0%)	0 (0%)	0 (0%)	
Lt	0 (0%)	1 (20%)	5 (25%)	
Bilateral	0 (0%)	7 (70%)	9 (45%)	
SVV				
Normal	20 (100%)	9 (90%)	17 (85%)	0.441
Abnormal				
Rt	0 (0%)	0 (0%)	1 (5%)	
Lt	0 (0%)	1 (10%)	2 (10%)	
SVH				
Normal	20 (100%)	5 (50%)	15 (75%)	0.019*
Abnormal				
Rt	0 (0%)	1 (10%)	2 (10%)	
Lt	0 (0%)	4 (40%)	3 (15%)	

*GIIa* study subgroup had OD with OH, *GIIb* study subgroup had OD without OH

*Significant at *P* < 0.05

In OD without OH study subgroup (IIb), 13 patients (65%) showed either bilateral or Left absent cVEMP waves. Moreover, 14 patients (70%) showed absent oVEMP waves either bilaterally or in the left ears. Three patients (15%) showed abnormal SVH values and five patients (25%) showed abnormal SVV values (Table 5).

The detectability of cVEMP, oVEMP waves, SVV and SVH were compared between the control and the two study subgroups. There were statistically significant differences of cVEMP, oVEMP and SVH test (*P* = 0.001, *P* = 0.001, *P* = 0.019), respectively. While there were no significant difference of SVV results (*P* = 0.441) between the three groups (Table 5).

Comparing latencies and amplitudes of P13, N23 and N10, P15 of cervical and ocular VEMP waves between the control and the two study subgroups were done using (ANOVA) and Post Hoc test. There were statistically significant differences of cVEMP waves P13, N23 latencies in the left ears only when comparing the control and OH study subgroup IIa (*P* = 0.003, *P* = 0.004), respectively. In addition, there were statistically significant differences of (P13–N23) amplitudes in left ears when comparing the control with OD without OH study subgroup IIb (*P* = 0.029). There was significant difference in N23 latency in the left ear only when comparing the two study subgroups (*P* = 0.049) (Table 6). While, there were no significant difference of oVEMP waves latencies or amplitudes between the three groups.

**Table 6:** Comparison of the cVEMPs wave latencies (in msec) and amplitudes (in μv) between control and study subgroups

c VEMP	Range	Mean ± SD	*F*. test	*P*. value	Post	Hoc test
Right P13 latency						
Control	10.7–18.5	13.82 ± 1.79	1.009	0.375	P1	0.269
GIIa	11.5–20.9	15.10 ± 3.90			P2	0.251
GIIb	11.9–19.2	14.84 ± 2.57			P3	0.830
Right N23 latency						
Control	17.2–24.3	20.80 ± 1.77	2.260	0.119	P1	0.063
GIIa	18.4–28.8	22.90 ± 3.51			P2	0.154
GIIb	18.7–26.6	22.02 ± 2.56			P3	0.456
P13–N23 amplitude						
Control	18.1–62.5	37.24 ± 12.85	1.703	0.196	P1	0.975
GIIa	15.9–71.1	37.45 ± 19.76			P2	0.089
GIIb	9.2–54.5	28.41 ± 13.33			P3	0.204
Left P13 latency						
Control	11.8–18.3	14.53 ± 1.78	5.418	0.010*	P1	0.003*
GIIa	14–21.4	17.58 ± 2.56			P2	0.264
GIIb	12.2–18.1	15.53 ± 2.17			P3	0.076
Left N23 latency						
Control	17.3–24.2	21.28 ± 1.85	4.953	0.014*	P1	0.004*
GIIa	21.3–29.4	24.30 ± 2.74			P2	0.470
GIIb	18.7–24.6	21.94 ± 2.03			P3	0.049*
P13–N23 amplitude						
Control	18.4–70.4	38.43 ± 13.47	2.985	0.045*	P1	0.365
GIIa	17.2–55.4	32.63 ± 15.38			P2	0.029*
GIIb	14.1–48.3	24.84 ± 11.94			P3	0.309

*GIIa* study subgroup had OD with OH, *GIIb* study subgroup had OD without OH, *P1* Control and GIIa, *P2* Control and GIIb, *P3* GIIa and GIIb

^*^Significant at *P* < 0.05

SVV and SVH results were compared between the three groups. Control and OH study subgroup IIa differed significantly in SVV and SVH values deviated to the left (*P* = 0.006, *P* = 0.015), respectively. There were no significant differnce between the control and OD without OH subgroup IIb or between the two study subgroups (Table 7).

**Table 7:** Comparison of SVV and SVH results between the control and the two study subgroups

	Range	Mean ± SD	*F*. test	*P*. value	Post	Hoc test
SVV RT						
Control	0.1–1.1	0.46 ± 0.26	2.193	0.123	P1	0.052
GIIa	0.2–1.9	0.92 ± 0.60			P2	0.310
GIIb	0–2.5	0.65 ± 0.75			P3	0.219
SVV LT						
Control	0–1.2	0.43 ± 0.28	4.177	0.021*	P1	0.006*
GIIa	0.1–2.9	1.12 ± 0.85			P2	0.137
GIIb	0–2.6	0.73 ± 0.72			P3	0.109
SVH RT						
Control	0.3–1.9	1.12 ± 0.52	0.463	0.632	P1	0.411
GIIa	0–2.3	1.34 ± 0.73			P2	0.916
GIIb	0–2.7	1.10 ± 0.76			P3	0.364
SVH LT						
Control	0.3–1.5	0.93 ± 0.36	3.248	0.048*	P1	0.015*
GIIa	0.1–2.8	1.62 ± 0.94			P2	0.206
GIIb	0–2.9	1.22 ± 0.82			P3	0.144

*GIIa* study subgroup had OD with OH, *GIIb* study subgroup had OD without OH, *P1* Control and GIIa, *P2* Control and GIIb, *P3* GIIa and GIIb

*Significant at *P* < 0.05

## Discussion

Orthostatic dizziness refers to dizziness or unsteadiness that is developed on rising from a sitting to a standing, or from lying to a sitting or standing position [2]. It is a common complaint in general practice [6].

The vestibular-autonomic reflexes include complex interactions and multiple pathways stimulated by vestibular activation, resulting in respiratory and cardiovascular (blood pressure and heart rate) changes through vestibulosympathetic reflex [7, 8]. Pliego et al. [9] reported significantly reduced heart rate in individuals exposed to transmastoid galvanic vestibular stimulation while in seated or standing positions. Evidence suggests that vestibular stimulation can entrain respiratory rhythm and alter both expiratory and inspiratory discharge signals [10, 11].

It was hypothesized that the vestibular system contributes in eliciting the required changes in blood pressure during movement and changes in posture [12]. On standing up, sympathetic vasomotor responses via baroreceptors triggers appropriate vasomotor and cardiomotor responses to maintain appropriate blood pressure. This mechanism is important to compensate for the change in body position and ensure adequate cerebral perfusion. Orthostatic dizziness is the commonly assumed presentation of vestibulo-sympathetic reflex dysfunction [13, 14].

Orthostatic/postural hypotension (OH) is the condition in which significant drop in systolic (≥ 20 mmHg) and/or diastolic (≥ 10 mmHg) blood pressure within 3 min after standing or during head up tilt test. It is strongly associated with OD, but it is not the only cause [2].

In our study, there were significant difference between the control group and the study group in systolic and diastolic blood pressure in standing positions. Ten patients (33.3%) of the study group were fulfilling the criteria of orthostatic hypotension.

In the controls, cVEMPs and oVEMP were successfully recorded from all subjects, while in the study group, 17 patients (56.7%) showed absent cVEMPs and 22 patients (73.3%) showed absent oVEMPs. These results were in agreement with those of Lin et al. [15] who studied cVEMP and o VEMP in 60 patients with OD. In that study, the authors demonstrated 30 patients (50%) had abnormal cVEMP responses and abnormal oVEMP in 24 patients (40%). Furthermore, the results of the current work were also close to those of Ahn et al. [16] who reported abnormal cVEMP in 60.2% of the OD subjects.

In the current study, it was noticed that the amplitude of P13–N23 of cVEMPs and N10–P15 of oVEMPs showed great variability. This agreed with results of Alpini et al. [17] and Murofushi et al. [18]. Those authors reported wide variability on P13–N23 amplitude that was detected intra-subjects and inter-subject in the trials most of the time.

There were statistically significant differences of P13 and N23 latencies and (P13–N23) amplitudes between control and study groups in the left ears only. The presence of a significant difference in the left ears only could be explained by two speculations. The first, is the asymmetric size of the left and right vertebral arteries as the main vascular supply of the vestibular system [19]. It was recorded that the left vessel is relatively larger than the right side [20]. This indicates that the blood flow in the two arteries likely differs. The blood flow is better in the smaller artery (the right side). Thus, the complications of impaired blood flow owing to OH are more likely to arise in the bigger artery, rather than the smaller artery.

The second speculation is that the dominant cerebral hemisphere might need more blood supply. The left dominant cerebral hemisphere could show more sensitivity to the generalized cerebral ischemia induced by OH [21]. This would produce vestibular dysfunction in the left side first. However, there could be individual differences in the anatomy of blood supply and vulnerability for the ischemic insults.

In the current study, all subjects in the controls had normal SVV and SVH. While in the study group, ten patients (33.3%) showed abnormal SVH values and 4 patients (13%) showed abnormal SSV results. In addition, both groups differed significantly in SVH values deviated to the left side.

The otolith is graviceptive organs, receives a signal of head tilt with respect to the gravitational vector and notifies the brain about rapid changes in posture. Otolith organs sends direct projections to caudal brainstem sites involved in the central regulation of respiratory and cardiovascular (blood pressure and heart rate) activity [22].

It was suggested that otolith organ dysfunction and impaired vestibulosympathetic reflex might cause the development of orthostatic dizziness [8, 23].

Our results indicate abnormal affection of otolith organ (both utricle and saccule) in these patients and significant relationship between OD and otolith dysfunction. Bogle [22] and Murofushi et al. [24] agreed with our results and they concluded that the majority of patients with OD have isolated otolith dysfunction.

In our research, ten subjects (33.3%) out of 30 fulfilled the criteria of OH. Accordingly, we further subdivided the study group into two subgroups based on their blood pressure testing into: subgroup (GIIa) who had OD with OH (10 patients) and Subgroup (GIIb) who had OD without OH (20 patients). To determine the association between otolith dysfunction and orthostatic dizziness with and without orthostatic hypotension.

In OH study subgroup, 40% showed bilateral absent cVEMP and 80% showed absent oVEMP either bilaterally or in the left ears. SVH values were abnormal in 50%. Furthermore, there were statistically significant differences of cVEMP waves latencies in the left ears only when compared with the control. In OD without OH study subgroup, 65% showed either bilateral or Left absent cVEMP, and 70% showed absent oVEMP waves either bilaterally or in the left ears.

So, according to these results both study subgroups (OD with and without OH) showed otolith dysfunction. This may be explained that although OD patients without OH did not fulfill the criteria of OH, there was statistically significant difference in the standing diastolic blood pressure between the control and this group (Table 4). In addition, the blood pressure measurements in this group tend to be less than the control group even if it did not reach significant values. Moreover, the vestibular sympathetic reflex regulates not only the blood pressure but also the heart rate and respiratory system [8, 10, 22]. However, the vestibular autonomic interaction is not completely understood. Therefore, more research with larger sample size is needed to study otolith function in patients with OD with and without OH with measurement of heart rate and respiratory changes between sitting and standing positions so appropriate recommendations and management can be efficiently addressed.

## Conclusions

Not all patients with orthostatic dizziness (OD) have orthostatic hypotension (OH). Otolith malfunction may be the cause of orthostatic dizziness (OD) in patients with or without orthostatic hypotension. More clinical research is needed to study role of otolith dysfunction in patients with OD without OH with larger sample size.

## Data Availability

Available when editor requests.
